# Zearalenone Exposure Disrupts Blood–Testis Barrier Integrity through Excessive Ca^2+^-Mediated Autophagy

**DOI:** 10.3390/toxins13120875

**Published:** 2021-12-08

**Authors:** Jinjin She, Nannan Feng, Wanglong Zheng, Hao Zheng, Peirong Cai, Hui Zou, Yan Yuan, Jianhong Gu, Zongping Liu, Jianchun Bian

**Affiliations:** 1 College of Veterinary Medicine, Yangzhou University, 12 Wenhui East Road, Yangzhou 225009, China; MX120190774@yzu.edu.cn (J.S.); DX120190150@yzu.edu.cn (N.F.); wanglongzheng@yzu.edu.cn (W.Z.); mx120180739@yzu.edu.cn (H.Z.); MX120170756@yzu.edu.cn (P.C.); zouhui@yzu.edu.cn (H.Z.); yuanyan@yzu.edu.cn (Y.Y.); jhgu@yzu.edu.cn (J.G.); liuzongping@yzu.edu.cn (Z.L.); 2 Jiangsu Co-Innovation Center for Prevention and Control of Important Animal Infectious Diseases and Zoonoses, Yangzhou 225009, China; 3 Joint International Research Laboratory of Agriculture and Agri-Product Safety of the Ministry of Education of China, Yangzhou University, Yangzhou 225009, China

**Keywords:** zearalenone, TM4 cells, blood–testis barrier, Ca^2+^, autophagy

## Abstract

Zearalenone (ZEA), a common mycotoxin in grains and animal feeds, has been associated with male reproductive disorders. However, the potential toxicity mechanism of ZEA is not fully understood. In this study, in vivo and in vitro models were used to explore the effects of ZEA on the blood–testis barrier (BTB) and related molecular mechanisms. First, male BALB/C mice were administered ZEA orally (40 mg/kg·bw) for 5–7 d. Sperm motility, testicular morphology, and expressions of BTB junction proteins and autophagy-related proteins were evaluated. In addition, TM4 cells (mouse Sertoli cells line) were used to delineate the molecular mechanisms that mediate the effects of ZEA on BTB. Our results demonstrated that ZEA exposure induced severe testicular damage in histomorphology and an ultrastructural, time-dependent decrease in the expression of blood–testis barrier junction-related proteins, accompanied by an increase in the expression of autophagy-related proteins. Additionally, similar to the in vitro results, the dose-dependent treatment of ZEA increased the level of cytoplasmic Ca^2+^ and the levels of the autophagy markers LC3-II and p62, in conjunction with a decrease in the BTB junction proteins occludin, claudin-11, and Cx43, with the dislocation of the gap junction protein Cx43. Meanwhile, inhibition of autophagy by CQ and 3-MA or inhibition of cytoplasmic Ca^2+^ by BAPTA-AM was sufficient to reduce the effects of ZEA on the TM4 cell BTB. To summarize, this study emphasizes the role of Ca^2+^-mediated autophagy in ZEA-induced BTB destruction, which deepens our understanding of the molecular mechanism of ZEA-induced male reproductive disorders.

## 1. Introduction

Zearalenone (ZEA), also known as the F-2 or RAL mycotoxin, is produced by fungi belonging to the *Fusarium* family, which show strong estrogenic activity [1,2]. ZEA has been proved to induce reproductive toxicity, immunotoxicity, genotoxicity, hepatotoxicity, and so forth [1,3,4]. As the molecular structure of ZEA is similar to that of estrogen [5], it is particularly harmful to the reproductive system. A previous study illustrated that the presence of ZEA in the diet of piglets caused significant increases in vulva size, the genital organ coefficient, and the level of certain immunoglobulins or hormones [6]. Similarly, a study conducted by Pang et al. [7,8] showed that long-term exposure to low concentrations of ZEA can cause disturbances in the reproductive capacity of male mice.

The blood–testis barrier (BTB) formed by Sertoli cells is a target of environmental toxicants [9]. It has been found that disorders of the intracellular environment and damage to Sertoli cell junction complex-related proteins (such as claudin-5, claudin-11, occludin, connexin-43, and ZO-1) are the main reasons for the destruction of BTB, which affects spermatogenesis and eventually leads to infertility [10]. Previous studies have shown that acute exposure to ZEA can reduce sperm motility and significantly reduce testosterone and estradiol levels. In addition, the activity of testicular marker enzymes and related BTB mRNA and protein expression are also significantly affected [11,12], indicating the toxic effects of ZEA on the male reproductive system. However, the underlying toxicity mechanisms are not entirely clear.

As an evolutionary conserved lysosomal catabolic mechanism, autophagy plays an important role in removing misfolded or aggregated proteins and in clearing damaged organelles. Excessive stimulation can overstimulate autophagy, interfere with cell protein metabolism, and disrupt the normal functioning of cells [13]. Autophagy includes multiple stages, such as nucleation and extension of phagocytic vesicles, maturation of autophagosomes, and degradation of lysosomes [14,15]. In recent years, autophagy has also been found to be involved in the degradation of connexins on the cell surface [16]. Our previous study showed that autophagy is involved in ZEA-induced cytoskeleton damage in Sertoli cells [17]. However, it is not clear whether autophagy is involved in ZEA-induced BTB destruction.

Ca^2+^ is an important secondary messenger that is essential for the survival of all higher organisms. [18]. Ca^2+^ is involved in multiple biological processes of cells, including cell proliferation, apoptosis, autophagy, hormone secretion, and gene regulation [19,20]. When cells are stimulated by extracellular stimulation, Ca^2+^ is released from the extracellular space or from intracellular Ca^2+^ stores [21]. Although there is no evidence that Ca^2+^ can directly break the BTB, many experimental studies suggest that the destruction of tight and gap junctions may involve intermediates activated by Ca^2+^, such as kinases (e.g., p38 mitogen-activated protein kinase (MAPK)) [22], phosphatases (e.g., calcineurin) [23], or protein kinase C (PKC) [24]. Researchers believe that Ca^2+^ has a positive and negative regulatory effect on autophagy under normal and stress conditions, respectively. Studies have shown that Ca^2+^ promotes autophagy through a variety of pathways, such as IP3R and beclin1 pathway and calmodulin-dependent protein kinase beta CaMKKβ–AMPK–mTOR pathway [25,26,27]. It is also believed that Ca^2+^ may inhibit autophagy through IP3R, BECLIN1-Bcl-2 complex, and AMPK-mTOR pathways [28,29]. In this study, we investigated how calcium-induced autophagy is involved in the regulation of testicular toxicity and BTB damage induced by ZEA.

## 2. Results

### 2.1. Effects of ZEA on Mice Organ Coefficient and Sperm Motility

**Figure 1:**
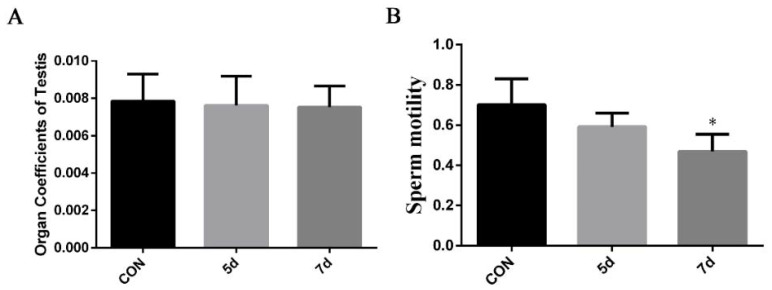
Effects of ZEA on mice organ coefficient and sperm motility. Mice were administered ZEA orally (40 mg/kg·bw) by gavage for 5 and 7 d. (**A**) Organ coefficient of testes. After ZEA exposure, testes were isolated and weighted and normalized to the bodyweight. (**B**) Sperm motility. The Integrated Semen Analysis System (ISAS) was used to analyze sperm motility. * *p* < 0.05, compared with the control group.

As shown in Figure 1A, compared with the control group, there was no significant difference in the organ coefficients of testes between the control group and the ZEA-treated group. However, a time-dependent decrease in sperm motility was observed in the ZEA-treated group (Figure 1B). These results suggest that ZEA induction affects mice spermatogenesis.

### 2.2. Histomorphological and Ultrastructural Changes in Testis after ZEA Exposure

**Figure 2:**
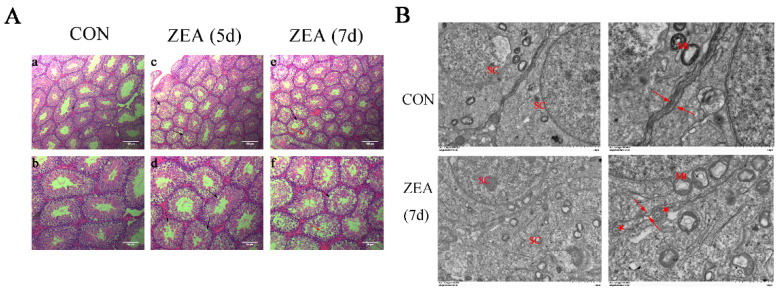
Histomorphological and ultrastructural changes in testis after ZEA exposure. (**A**) Testicular histomorphological changes in mice after treatment with ZEA. The morphologies of spermatogenic cells and Sertoli cells were normal, and elongated spermatids can be seen in the seminiferous tubule in the corn-oil-treated group (**a**,**b**); compared with the corn-oil-treated group, after 5 d of ZEA exposure, the testes of mice showed obvious thinning of the seminiferous lumen, loose seminiferous epithelium, and vacuolar changes between cells (black arrow, (**c**,**d**)); after 7 d of ZEA exposure, the testes showed sloughing of spermatogenic cells into the lumen (red arrow, (**e**,**f**)). For the upper panel, scale bar = 100 μm. For the lower panel, scale bar = 50 μm. (**B**) Ultrastructural changes in testis after 7 d of ZEA exposure. Compared with the control group, after 7 d of ZEA exposure, mitochondrial swelling and degeneration, destroyed BTB, and autophagosome accumulation were observed in testis. For the left panel, scale bar = 2 μm. For the right panel, scale bar = 1 μm. The red arrows indicate BTB; Mt indicates mitochondrial.

Subsequently, we examined the histological morphology of the testis after ZEA exposure through hematoxylin and eosin (H&E) staining. As shown in Figure 2A, compared with the control group, after 5 d of ZEA treatment, the testes of mice showed obvious thinning of the seminiferous lumen, loose seminiferous epithelium, and vacuolar changes among cells. After 7 d of ZEA treatment, sloughing of spermatogenic cells into the lumen was observed. Then, we conducted transmission electron microscopy on the testes of the mice in the control group and those in the 7 d ZEA treatment group. After ZEA exposure, mitochondrial swelling and degeneration, destroyed BTB, and autophagosome accumulation were observed in the testes (Figure 2B).

### 2.3. Effects of ZEA on the Expressions of BTB and Autophagy-Related Regulating Proteins in Mice Testes

**Figure 3:**
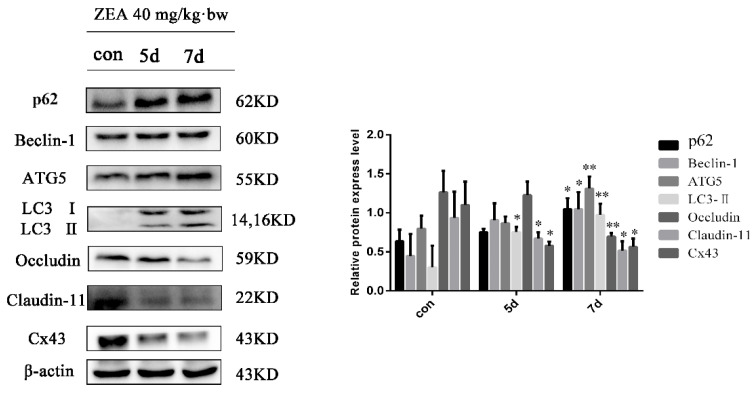
Effects of ZEA on the expressions of BTB and autophagy-related regulating proteins in mice testes. Male adult mice were administered ZEA orally (40 mg/kg·bw) by gavage for 5 and 7 d. After exposure, the expressions of p62, Beclin-1, ATG5, LC3, occludin, claudin-11, and Cx43 were evaluated. * *p* < 0.05 and ** *p* < 0.01, compared with the control group.

Based on the above models for mice, the effects of ZEA on BTB-related protein expressions in testes were evaluated. As shown in Figure 3, similar to its effects on BTB, ZEA increased the autophagy-related proteins p62, Beclin-1, ATG5, and LC3 and decreased the BTB-related junction proteins occludin, claudin-11, and Cx43 in a time-dependent manner. These results suggest that autophagy may be involved in ZEA-induced BTB disruption.

### 2.4. Effects of ZEA on the Expression of BTB-Related Regulating Proteins in TM4 Cells

**Figure 4:**
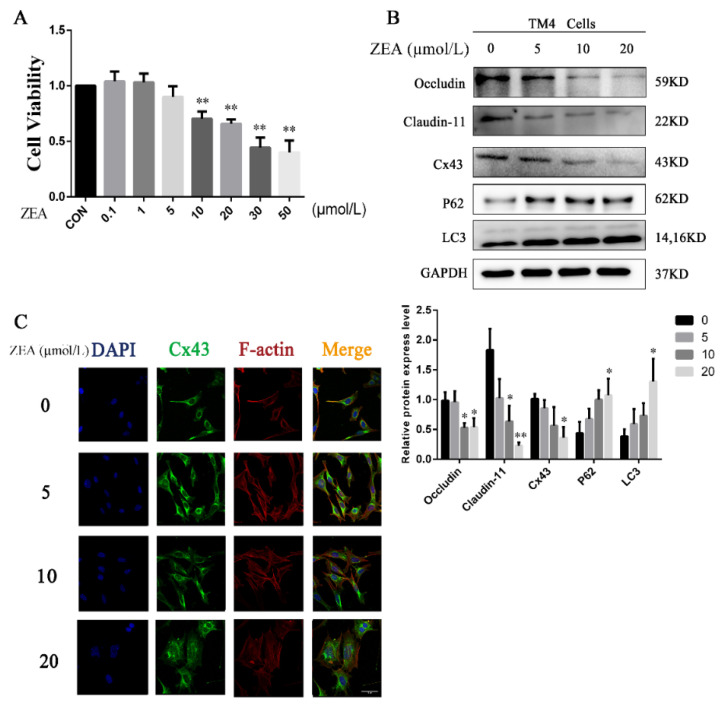
Effects of ZEA on the expression of BTB-related regulating proteins in TM4 cells. (**A**) Effect of ZEA on the viability of TM4 cells. (**B**) TM4 cells were treated with ZEA at concentrations of 0, 5, 10, and 20 μmol/L for 24 h. The expressions of occludin, claudin-11, Cx43, p62, and LC3 were estimated by immunoblot analysis. * *p* < 0.05 and ** *p* < 0.01, compared with the control group. (**C**) Localization of Cx43 in BTB after the exposure of TM4 cells to ZEA (20 μmol/L) for 24 h. TM4 cell nuclear (blue), Cx43 (green), F-actin (red), scale bar = 20 μm.

As shown in Figure 4A, the viability of TM4 cells decreased with an increase in the ZEA concentration, indicating the toxic effect of ZEA on TM4 cells. Sertoli cells are integral for maintaining the integrity of BTB. Therefore, to confirm the in vivo results and test whether these BTB-related regulating proteins are involved in ZEA-induced Sertoli cell BTB disruption, the expressions of p62, LC3, junction protein occludin, claudin-11, and Cx43 were evaluated for TM4 cells. As shown in Figure 4B, similar to the in vivo results, an increase was observed in the expressions of the autophagy-related proteins p62 and LC3, in conjunction with a decrease in the BTB-related junction protein occludin, claudin-11, and Cx43 in TM4 cells in a dose dependent manner with ZEA. In addition, Figure 4C demonstrates that the localization of Cx43 at the interface of TM4 cells significantly decreased. The observed cytoskeleton fracture by ZEA suggests that Cx43 may enter the cell to induce degradation.

### 2.5. Effect of Autophagy on the Damage of BTB-Related Protein in TM4 Cells Induced by ZEA

**Figure 5:**
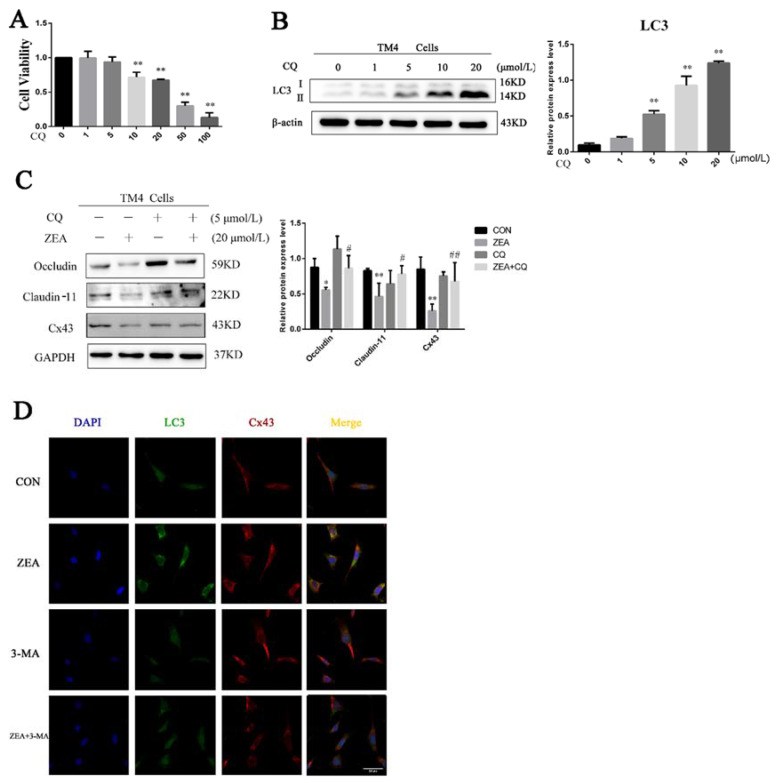
Effect of autophagy on the damage of BTB-related protein in TM4 cells induced by ZEA. (**A**) Effect of CQ on the viability of TM4 cells. (**B**) TM4 cells treated with different concentrations of CQ for 24 h; LC3 was detected by Western blot analysis. * *p* < 0.05 and ** *p* < 0.01, compared with the control group. (**C**) Expressions of occludin, claudin-11, and Cx43 were assayed after TM4 cells were pretreated with or without CQ for 0.5 h, and then treated with ZEA for 24 h. * *p* < 0.05 and ** *p* < 0.01, compared with the control group; # *p* < 0.05 and ## *p* < 0.01, compared with the ZEA-treated group. (**D**) Localization for LC3 punctate staining and Cx43 in BTB for TM4 cells exposed to 20 μmol/L ZEA for 24 h with or without 3-MA (5 mmol/L) pretreatment. TM4 cell nuclear (blue), LC3 (green), Cx43 (red), scale bar = 20 μm.

In order to determine the relationship of autophagy in this pathological process, the regulation of BTB-related proteins in TM4 cells was evaluated using CQ (a classical lysosome inhibitor). The concentration of CQ was selected as 5 μmol/L by CCK8 and Western blotting (Figure 5A,B). As shown in Figure 5C, the expressions of occludin, claudin-11, and Cx43 in the CQ-and-ZEA-combined treatment group were significantly higher than those in the ZEA-only treatment group. In addition, after TM4 cells were treated with ZEA and 3-MA for 24 h, LC3-II fluorescence was evenly distributed in the cytoplasm of TM4 cells in the control group and the group with 3-MA treatment alone, no obvious fluorescent autophagosome structure was observed, and Cx43 was evenly distributed on the cell membrane. In contrast, ZEA-treated fluorescent LC3-II autophagosomes significantly increased, and Cx43 was no longer evenly present around the cells, indicating that Cx43 may enter the cell and cause degradation through autophagy. After the inhibition of autophagy by 3-MA-combined treatment, it was found that fluorescent autophagosomes significantly decreased, and the localization of Cx43 was normal (Figure 5D).

### 2.6. Effect of Ca^2+^-Mediated Autophagy on the Damage to BTB-Related Proteins in TM4 Cells Induced by ZEA

**Figure 6:**
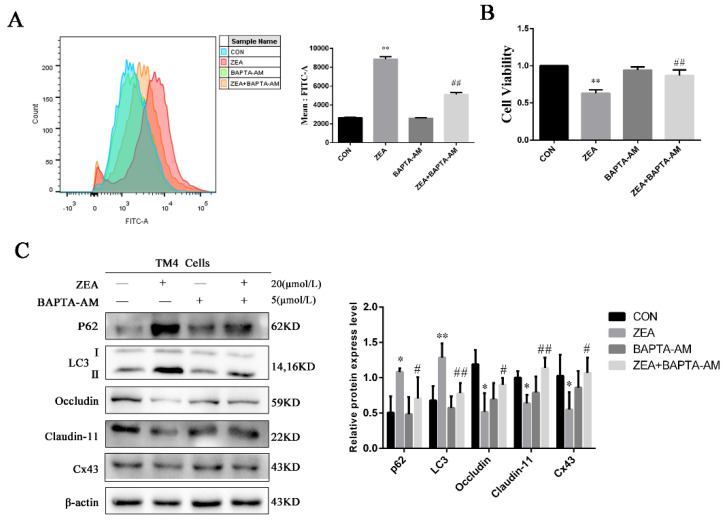
Effect of Ca^2+^-mediated autophagy on the damage to BTB-related proteins in TM4 cells induced by ZEA. (**A**) TM4 cells were treated with 5 μmol/L BAPTA-AM for 30 min, and then treated with 20 μmol/L ZEA for 24 h; flow cytometry detected intercellular levels of Ca^2+^. (**B**) Effect of ZEA and BAPTA-AM on the viability of TM4 cells. (**C**) Expression levels of p62, LC3, occludin, claudin-11, and Cx43 using Western blot analysis. TM4 cells were exposed to 20 μmol/L ZEA for 24 h with or without BAPTA-AM pretreatment (* *p* < 0.05 and ** *p* < 0.01, compared with the control group; # *p* < 0.05 and ## *p* < 0.01, compared with the ZEA-treated groups).

BAPTA-AM is a specific chelator of Ca^2+^. After pretreatment with 5 μmol/L BAPTA-AM, cells were treated with 20 μmol/L ZEA for 24 h. Changes in Ca^2+^ were detected using flow cytometry, as shown in Figure 6A. BAPTA-AM can significantly reduce the increase in intracellular calcium induced by ZEA. Apart from this, treatment with BAPTA-AM significantly inhibited the decrease in cell viability induced by ZEA (Figure 6B). These results strongly suggest that BAPTA-AM can effectively mitigate the cytotoxic damage caused by ZEA. After treatment with ZEA and BAPTA-AM, the expression of autophagy and BTB-related proteins was detected by Western blotting. As shown in Figure 6C, BAPTA-AM can effectively reduce the increase in the autophagic proteins p62 and LC3 caused by ZEA and increase the expressions of the BTB-related proteins occludin, claudin-11, and Cx43. These findings demonstrate that Ca^2+^-mediated autophagy may be involved in ZEA-induced BTB disruption.

## 3. Discussion

Zearalenone is present in a wide range of human foods, and studies have shown that zearalenone can cause male sterility and neurological dysfunction [30]. The purpose of this study was to investigate the effects of ZEA on BTB and the related in vivo and in vitro molecular mechanisms. In order to establish an in vivo model of ZEA-induced male reproductive injury, mice were administered ZEA orally at a dosage of 40 mg/kg·bw by gavage for 5 and 7 d according to previous reports to simulate a high-exposure scenario [12,31]. Reportedly, the contamination of ZEA in grains and animal feeds can range from 4 to 11,192 µg/kg [32]. Furthermore, the dose for the lowest observed effect level for developmental and reproductive toxicity has been reported to be approximately 20 mg/kg·bw in rodents [33]. Considering the sensitivity of pigs to ZEA [34] and the differences between rodents, pigs, and ruminants, we chose a ZEA dosage of 40 mg/kg·bw and implemented it in the present study. Our data show that ZEA exposure decreased sperm motility. Hyperchromatic nuclei were found in the lumen of the ZEA treatment group, indicating that spermatogenic cells fell into the lumen. Meanwhile, we observed mitochondrial swelling and degeneration, destroyed BTB, and autophagosome accumulation in the testis after ZEA exposure, which is supported by previous studies [31,35], indicating that the mouse-based model of ZEA-induced male reproductive injury is valid. The reason for the spermatogenesis impairment in immature testis after ZEA exposure remains unclear. BTB is a well-known prerequisite for spermatogenesis. When junctional proteins are abnormally expressed in Sertoli cells, spermatogenic cells do not show differentiation or meiosis, resulting in infertility [36,37,38]. We also found that autophagic proteins increased, and BTB-related proteins decreased with an increase in ZEA exposure time, indicating that BTB damage caused by ZEA is an important factor for the decrease in sperm motility, and autophagy may be involved in the regulation of BTB by ZEA.

CQ is a classical lysosome inhibitor, which inhibits the binding of lysosomes and autophagosomes by inducing the disintegration of the Golgi matrix and nucleosome/lysosome system and blocking the autophagy pathway downstream [39]. Our study found that CQ combined with ZEA can reverse the downregulation of occludin, claudin-11, and Cx43 proteins caused by ZEA. Autophagy is one of the most important degradation pathways in intracellular protein metabolism. Studies have found that external stimuli can promote the endocytosis and degradation of membrane surface proteins by activating the autophagy pathways of epithelial cells: nano-alumina can activate autophagy and mitochondrial damage in brain endothelial cells, thereby downregulating the expression of the tight junction proteins occludin and claudin-5. Pretreatment with Warman penicillin, an inhibitor of phagocytic pathways, can significantly alleviate the downregulation of connexin caused by alumina [40]. In addition, after glucose and oxygen deprivation, mouse brain microvascular endothelial cells (bEND3) can enter the cytoplasm through caveolin-1 targeting the membrane surface connexin claudin-5 and enter LC3-II autophagosomes for degradation. The inhibition of autophagy or blocking of the binding of lysosomes to autophagosomes could prevent the degradation of claudin-5 [41]. In addition, we treated TM4 cells with 3-MA, an inhibitor of the upstream PI3K/Beclin-1/Atg13 complex of autophagy [42], which inhibited autophagy. Moreover, we detected the colocalization of LC3-II and Cx43 by immunofluorescence and found that ZEA can transfer the membrane protein Cx43 to the cytoplasm and cause degradation by activating autophagy pathways, whereas the inhibition of autophagy by 3-MA could significantly inhibit the degradation of Cx43. Interestingly, the results were consistent with those of previous studies that reported that 3-MA could alleviate the increase in intestinal permeability and the decrease and recombination of TJ proteins induced by burns [43]. Overall, these results suggest that autophagy may be involved in ZEA-induced BTB disruption.

Intracellular free Ca^2+^ ions are important signaling molecules in cells, participating in various physiological and pathological processes [44]. Free Ca^2+^ ions in the cell are input from the outside of the cell mainly through the Na^+^/Ca^2+^ exchanger, the plasma membrane Ca^2+^-ATPase (PMCA). An increase in the intracellular free Ca^2+^ concentration is mainly caused by extracellular Ca^2+^ ions through the voltage-gated ion channel (VGCC). Ca stores regulate the Ca^2+^ receptor-operated channels (ROCs) into cells, and intracellular Ca stores release Ca^2+^ ions into cells through IP3 and ryanodine receptor Ca channels [45,46,47]. Many studies have shown that an increase in intracellular Ca^2+^ concentrations is the main cause of apoptosis and even death [48,49]. Our results showed that ZEA could increase the concentration of intracellular Ca^2+^ in a dose-dependent manner, and the decrease in cell viability induced by ZEA can be effectively alleviated by adding the intracellular Ca integrator BAPTA-AM. These results show that a Ca^2+^ overload in TM4 cells may be caused by the release of intracellular Ca stores by ZEA, which is consistent with the results of our previous study [27]. Our study found that BAPTA-AM combined with ZEA can reverse the upregulation of LC3-II and p62 proteins induced by ZEA, indicating that the increase in intracellular Ca may be involved in autophagy induced by ZEA. Interestingly, when we combined BAPTA-AM with ZEA, we found that BAPTA-AM could alleviate the downregulation of tight and gap junction-related proteins induced by ZEA. These results strongly suggest that Ca^2+^ can mediate the downregulation of these proteins in this pathological process. Indeed, Sertoli cell BTB-related junction proteins are reportedly the substrates that are indirectly regulated by Ca^2+^ [50,51]. Overall, our results strongly suggest that Ca^2+^-mediated autophagy may be involved in ZEA-induced BTB disruption, which could contribute to a better understanding of potential molecular mechanisms of ZEA-induced reproductive toxicity, and possibly a novel potential target for preventive and/or therapeutic treatments.

## 4. Conclusions

**Figure 7:**
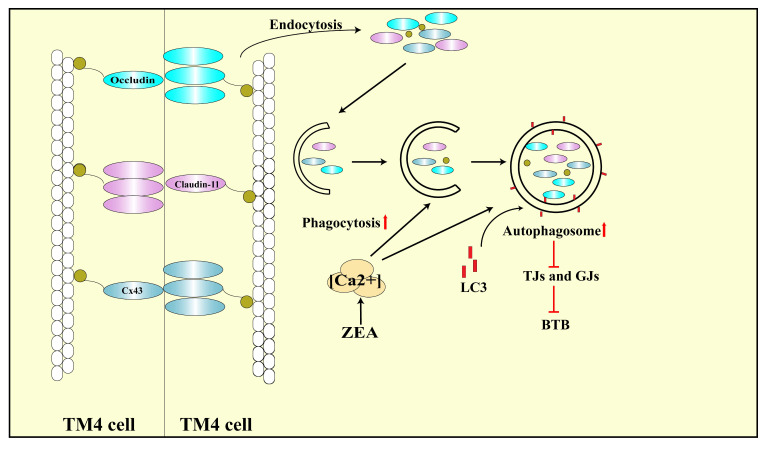
Schematic diagram of the proposed mechanisms of Ca^2+^-mediated autophagy mediating ZEA-induced BTB disruption.

In summary, our study confirmed that ZEA disrupted BTB by increasing the concentration of Ca^2+^ in the cytoplasm and inducing the accumulation of autophagosomes (Figure 7). Thus, the substantial activation of Ca^2+^, elevation in the expression of autophagy-related proteins, and decrease in dislocation of tight and gap junction-related proteins provide a novel molecular mechanism for ZEA-induced BTB disruption and related male reproductive disorders.

## 5. Materials and Methods

### 5.1. Chemicals and Reagent

ZEA (purity of ≥99.0%) was purchased from Sigma-Aldrich (St. Louis, MO, USA). DMEM/F-12 medium and fetal bovine serum (FBS) were obtained from Gibco BRL (USA). Chloroquine (CQ), rapamycin (Rap), 3-methyladenine (3-MA), and BAPTA-AM were obtained from MedChemexpress (St. Louis, MO, USA). BCA protein assay kit, Fluo-3AM, and DAPI were obtained from Beyotime Institute of Biotechnology (Shanghai, China). The antibodies occludin, claudin-11, and ATG5 were obtained from Abcam Corp (Cambridge, MA, USA). The antibodies p62, Beclin-1, GAPDH, and β-actin were purchased from Cell Signaling Technology Corp (Boston, MA, USA). LC3 and connexin 43 (Cx43) were purchased from Sigma-Aldrich (St. Louis, MO, USA). All chemicals and reagents were analytical grade.

### 5.2. Animal and Treatment

Eighteen adult male BALB/C mice (6 weeks old, from the Comparative Medicine Center of Yangzhou University, China) were housed in a room with a controlled temperature (25 ± 1 °C) and 12 /12 h light/dark cycle. The mice were given free to access to drinking water and mouse chow. The care and use of the animals during this experiment followed the guidelines of the Animal Care and Welfare Committee of Yangzhou University. The license number of the experimental animals was SYXK (Su) 2021-0026, and the time was 26 March 2021 to 25 March 2026. In this study, mice were randomly divided into three groups, and ZEA at a dose of 40 mg/kg·/bw was administered orally by gavage for 5 and 7 d according to previous reports, which was conducted to simulate the high-exposure scenario [12,30]. For the control group, mice were given the same volume of corn oil. After final exposure, the mice were humanely euthanized, and the testes were isolated and weighted.

### 5.3. Sperm Motility

Freshly isolated epididymis was briefly placed in 2 mL normal saline. Six deep cuts were made in each cauda using microscissors to release sperm into the media. The sperm suspension was incubated in a water bath for 20 min; after 10 min of incubation (at 37 °C), a drop of sperm suspension was placed in a preheated blood cell count board. The Integrated Semen Analysis System (ISAS, Paterna, Spain) was used to analyze sperm motility.

### 5.4. Histopathological Observation of the Testes

Simply, testes were fixed in 4% paraformaldehyde for 24 h and then washed in running water for 6 h, dehydrated with ethanol gradient embedded in paraffin, and sectioned. Hematoxylin and eosin staining was used for routine staining. Images were observed under a Leica microscope.

### 5.5. Ultrastructure Observation of the Testes

The experiment was conducted under the guidance of standard technology in the pathology electron microscope equipment of Yangzhou University.

### 5.6. Cell Culture

TM4 cells (mouse Sertoli cell line) were purchased from the American Type Culture Collection (ATCC, Rockefeller, MD, USA). Cells were cultivated in DMEM/F12 and added to a 10% FBS 1% of penicillin and 1 mM glutamine medium and cultured at 37 °C with 5% CO_2_.

### 5.7. Cell Proliferation Assay

After the cells were plated at a density of 1 × 104 per well in a 96-well plate. Control wells, blank wells, and experimental wells were set separately, with 6 replicates in each group. CCK-8 solution (10 μM) was added to each well, the culture plate was placed in an incubator for 2–4 h, and the absorbance was determined at 450 nm.

### 5.8. Measurement of Intracellular Ca^2+^

Immediately after the cells were treated according to the processes mentioned above, they were collected and induced with Fluo-3AM at 37 °C in the dark for 30 min. The cells were monitored using a flow cytometer at an excitation wavelength of 488 nm.

### 5.9. Western Blotting Analysis

Freshly isolated testis and TM4 cells were lysed, and the concentration of protein was measured using a BCA protein assay kit, followed by Western blot at a concentration of 10–30 μg. After electrophoresis, proteins were transferred to a polyvinylidene fluoride membrane and incubated in 5% skim milk for 2 h. The membranes were incubated with primary antibodies at 4 °C for 12 h, washed 5 times with TBST, and then incubated with HRP-linked secondary antibodies for 2 h. After washing 5 times with TBST, the protein signals were detected using an ECL detection system and analyzed with the ImageJ software.

### 5.10. Immunofluorescence Analysis

TM4 cells were seeded in a 24-well plate. After treatment, the cells were washed and fixed in 4% paraformaldehyde at 4 °C for 30 min. After washing, they were treated with 0.5% Triton X-100 at room temperature for 15 min and then incubated with 5% BSA sealing solution for 1 h. The cells were then incubated overnight with the corresponding primary antibody. After being washed, the cells were then incubated with fluorescent-labelled secondary antibodies for 1 h in the dark. The localization of Cx43 and LC3 in TM4 cells was detected via confocal fluorescence microscopy.

### 5.11. Statistical Analysis

SPSS 22.0 software was used to analyze the quantitative data by one-way analysis of variance (ANOVA). The level of statistical significance was set at *p* < 0.05.

## Data Availability

Not applicable.

## References

[1] Zinedine A., Soriano J.M., Molto J.C., Manes J. (2007). Review on the toxicity, occurrence, metabolism, detoxification, regulations and intake of zearalenone: An oestrogenic mycotoxin. Food Chem. Toxicol..

[2] Rogowska A., Pomastowski P., Sagandykova G., Buszewski B. (2019). Zearalenone and its metabolites: Effect on human health, metabolism and neutralisation methods. Toxicon.

[3] Lioi M.B., Santoro A., Barbieri R., Salzano S., Ursini M.V. (2004). Ochratoxin A and zearalenone: A comparative study on genotoxic effects and cell death induced in bovine lymphocytes. Mutat. Res..

[4] Skiepko N., Przybylska-Gornowicz B., Gajecka M., Gajecki M., Lewczuk B. (2020). Effects of Deoxynivalenol and Zearalenone on the Histology and Ultrastructure of Pig Liver. Toxins.

[5] Takemura H., Shim J.Y., Sayama K., Tsubura A., Zhu B.T., Shimoi K. (2007). Characterization of the estrogenic activities of zearalenone and zeranol in vivo and in vitro. J. Steroid Biochem. Mol. Biol..

[6] Su Y., Sun Y., Ju D., Chang S., Shi B., Shan A. (2018). The detoxification effect of vitamin C on zearalenone toxicity in piglets. Ecotoxicol. Environ. Saf..

[7] Pang J., Zhou Q., Sun X., Li L., Zhou B., Zeng F., Zhao Y., Shen W., Sun Z. (2017). Effect of low-dose zearalenone exposure on reproductive capacity of male mice. Toxicol. Appl. Pharm..

[8] Crisostomo L., Alves M.G., Gorga A., Sousa M., Riera M.F., Galardo M.N., Meroni S.B., Oliveira P.F. (2018). Molecular Mechanisms and Signaling Pathways Involved in the Nutritional Support of Spermatogenesis by Sertoli Cells. Methods Mol. Biol..

[9] Gao Y., Mruk D.D., Cheng C.Y. (2015). Sertoli cells are the target of environmental toxicants in the testis—A mechanistic and therapeutic insight. Expert Opin. Ther. Targets.

[10] Wang X.N., Li Z.S., Ren Y., Jiang T., Wang Y.Q., Chen M., Zhang J., Hao J.X., Wang Y.B., Sha R.N. (2013). The Wilms tumor gene, Wt1, is critical for mouse spermatogenesis via regulation of sertoli cell polarity and is associated with non-obstructive azoospermia in humans. PLoS Genet..

[11] Yang S., Gong P., Pan J., Wang N., Tong J., Wang M., Long M., Li P., He J. (2019). Pediococcus pentosaceus xy46 Can Absorb Zearalenone and Alleviate its Toxicity to the Reproductive Systems of Male Mice. Microorganisms.

[12] Long M., Yang S., Dong S., Chen X., Zhang Y., He J. (2017). Characterization of semen quality, testicular marker enzyme activities and gene expression changes in the blood testis barrier of Kunming mice following acute exposure to zearalenone. Environ. Sci. Pollut. Res. Int..

[13] Glick D., Barth S., Macleod K.F. (2010). Autophagy: Cellular and molecular mechanisms. J. Pathol..

[14] Nakamura S., Yoshimori T. (2017). New insights into autophagosome-lysosome fusion. J. Cell Sci..

[15] Napoletano F., Baron O., Vandenabeele P., Mollereau B., Fanto M. (2019). Intersections between Regulated Cell Death and Autophagy. Trends Cell Biol..

[16] Fong J.T., Kells R.M., Gumpert A.M., Marzillier J.Y., Davidson M.W., Falk M.M. (2012). Internalized gap junctions are degraded by autophagy. Autophagy.

[17] Zheng W., Wang B., Si M., Zou H., Song R., Gu J., Yuan Y., Liu X., Zhu G., Bai J. (2018). Zearalenone altered the cytoskeletal structure via ER stress- autophagy- oxidative stress pathway in mouse TM4 Sertoli cells. Sci. Rep..

[18] Harr M.W., Distelhorst C.W. (2010). Apoptosis and autophagy: Decoding calcium signals that mediate life or death. Cold Spring Harb. Perspect. Biol..

[19] Patergnani S., Danese A., Bouhamida E., Aguiari G., Previati M., Pinton P., Giorgi C. (2020). Various Aspects of Calcium Signaling in the Regulation of Apoptosis, Autophagy, Cell Proliferation, and Cancer. Int. J. Mol. Sci..

[20] Clapham D.E. (2007). Calcium signaling. Cell.

[21] Pinton P., Giorgi C., Siviero R., Zecchini E., Rizzuto R. (2008). Calcium and apoptosis: ER-mitochondria Ca2+ transfer in the control of apoptosis. Oncogene.

[22] Mu D., Zhang W., Chu D., Liu T., Xie Y., Fu E., Jin F. (2008). The role of calcium, P38 MAPK in dihydroartemisinin-induced apoptosis of lung cancer PC-14 cells. Cancer Chemother. Pharm..

[23] El-Hashash A.H.K., Turcatel G., Varma S., Berika M., Al Alam D., Warburton D. (2017). Retraction: Eya1 protein phosphatase regulates tight junction formation in lung distal epithelium. J. Cell Sci..

[24] Long A.C., Colitz C.M., Bomser J.A. (2007). Regulation of gap junction intercellular communication in primary canine lens epithelial cells: Role of protein kinase C. Curr. Eye Res..

[25] Chen X., Li M., Chen D., Gao W., Guan J.L., Komatsu M., Yin X.M. (2012). Autophagy induced by calcium phosphate precipitates involves endoplasmic reticulum membranes in autophagosome biogenesis. PLoS ONE.

[26] Xia H.G., Zhang L., Chen G., Zhang T., Liu J., Jin M., Ma X., Ma D., Yuan J. (2010). Control of basal autophagy by calpain1 mediated cleavage of ATG5. Autophagy.

[27] Feng N., Wang B., Cai P., Zheng W., Zou H., Gu J., Yuan Y., Liu X., Liu Z., Bian J. (2020). ZEA-induced autophagy in TM4 cells was mediated by the release of Ca(2+) activates CaMKKbeta-AMPK signaling pathway in the endoplasmic reticulum. Toxicol. Lett..

[28] Decuypere J.P., Bultynck G., Parys J.B. (2011). A dual role for Ca(2+) in autophagy regulation. Cell Calcium.

[29] Cardenas C., Foskett J.K. (2012). Mitochondrial Ca(2+) signals in autophagy. Cell Calcium.

[30] Kowalska K., Habrowska-Gorczynska D.E., Piastowska-Ciesielska A.W. (2016). Zearalenone as an endocrine disruptor in humans. Environ. Toxicol. Pharmacol..

[31] Long M., Yang S., Wang Y., Li P., Zhang Y., Dong S., Chen X., Guo J., He J., Gao Z. (2016). The Protective Effect of Selenium on Chronic Zearalenone-Induced Reproductive System Damage in Male Mice. Molecules.

[32] Kovalsky P., Kos G., Nahrer K., Schwab C., Jenkins T., Schatzmayr G., Sulyok M., Krska R. (2016). Co-Occurrence of Regulated, Masked and Emerging Mycotoxins and Secondary Metabolites in Finished Feed and Maize-An Extensive Survey. Toxins.

[33] Gao X., Xiao Z., Li C., Zhang J., Zhu L., Sun L., Zhang N., Khalil M.M., Rajput S.A., Qi D. (2018). Prenatal exposure to zearalenone disrupts reproductive potential and development via hormone-related genes in male rats. Food Chem. Toxicol..

[34] Rai A., Das M., Tripathi A. (2020). Occurrence and toxicity of a fusarium mycotoxin, zearalenone. Crit. Rev. Food Sci. Nutr..

[35] Yang D., Jiang X., Sun J., Li X., Li X., Jiao R., Peng Z., Li Y., Bai W. (2018). Toxic effects of zearalenone on gametogenesis and embryonic development: A molecular point of review. Food Chem. Toxicol..

[36] Sridharan S., Brehm R., Bergmann M., Cooke P.S. (2007). Role of connexin 43 in Sertoli cells of testis. Ann. N. Y. Acad. Sci..

[37] Noelke J., Wistuba J., Damm O.S., Fietz D., Gerber J., Gaehle M., Brehm R. (2015). A Sertoli cell-specific connexin43 knockout leads to altered interstitial connexin expression and increased Leydig cell numbers. Cell Tissue Res..

[38] Lie P.P., Cheng C.Y., Mruk D.D. (2011). The biology of the desmosome-like junction a versatile anchoring junction and signal transducer in the seminiferous epithelium. Int. Rev. Cell Mol. Biol..

[39] Mauthe M., Orhon I., Rocchi C., Zhou X., Luhr M., Hijlkema K.J., Coppes R.P., Engedal N., Mari M., Reggiori F. (2018). Chloroquine inhibits autophagic flux by decreasing autophagosome-lysosome fusion. Autophagy.

[40] Chen L., Zhang B., Toborek M. (2013). Autophagy is involved in nanoalumina-induced cerebrovascular toxicity. Nanomedicine.

[41] Liu J., Weaver J., Jin X., Zhang Y., Xu J., Liu K.J., Li W., Liu W. (2016). Nitric Oxide Interacts with Caveolin-1 to Facilitate Autophagy-Lysosome-Mediated Claudin-5 Degradation in Oxygen-Glucose Deprivation-Treated Endothelial Cells. Mol. Neurobiol..

[42] Wu Y., Wang X., Guo H., Zhang B., Zhang X.B., Shi Z.J., Yu L. (2013). Synthesis and screening of 3-MA derivatives for autophagy inhibitors. Autophagy.

[43] Huang Y., Wang Y., Feng Y., Wang P., He X., Ren H., Wang F. (2019). Role of Endoplasmic Reticulum Stress-Autophagy Axis in Severe Burn-Induced Intestinal Tight Junction Barrier Dysfunction in Mice. Front. Physiol..

[44] Bootman M.D., Collins T.J., Peppiatt C.M., Prothero L.S., MacKenzie L., De Smet P., Travers M., Tovey S.C., Seo J.T., Berridge M.J. (2001). Calcium signaling—An overview. Semin. Cell Dev. Biol..

[45] Kumar V.S.S., Gopalakrishnan A., Naziroglu M., Rajanikant G.K. (2014). Calcium Ion—The Key Player in Cerebral Ischemia. Curr. Med. Chem..

[46] Berridge M.J., Bootman M.D., Roderick H.L. (2003). Calcium signalling: Dynamics, homeostasis and remodelling. Nat. Rev. Mol. Cell Biol..

[47] Gurkoff G., Shahlaie K., Lyeth B., Berman R. (2013). Voltage-gated calcium channel antagonists and traumatic brain injury. Pharmaceuticals.

[48] Xu S., Pi H., Chen Y., Zhang N., Guo P., Lu Y., He M., Xie J., Zhong M., Zhang Y. (2013). Cadmium induced Drp1-dependent mitochondrial fragmentation by disturbing calcium homeostasis in its hepatotoxicity. Cell Death Dis..

[49] Bauer T.M., Murphy E. (2020). Role of Mitochondrial Calcium and the Permeability Transition Pore in Regulating Cell Death. Circ. Res..

[50] De Vuyst E., Wang N., Decrock E., De Bock M., Vinken M., Van Moorhem M., Lai C., Culot M., Rogiers V., Cecchelli R. (2009). Ca(2+) regulation of connexin 43 hemichannels in C6 glioma and glial cells. Cell Calcium.

[51] De Bock M., Wang N., Decrock E., Bol M., Gadicherla A.K., Culot M., Cecchelli R., Bultynck G., Leybaert L. (2013). Endothelial calcium dynamics, connexin channels and blood-brain barrier function. Prog. Neurobiol..

